# An unusual cause of chronic diarrhea: plastron appendicitis 

**Published:** 2019

**Authors:** Bilal Katipoğlu, Gökhan Yırgın, Burak Furkan Demir, İhsan Ateş

**Affiliations:** 1 *Ankara Numune Training and Research Hospital, Department of Internal Medicine, Ankara, Turkey*; 2 *Haseki Training and Research Hospital, Department of Internal Medicine, İstanbul, Turkey *

**Keywords:** Plastron appendicitis, chronic diarrhea, appendicitis

## Abstract

Chronic diarrhea is defined as diarrhea that lasts longer than four weeks. Etiology of chronic diarrhea includes inflammatory bowel disease, malabsorption syndromes, irritable bowel disease, chronic parasitic infections, bacterial toxins, drugs and motility disorders. Plastron appendicitis is an abscess formation that occurs when the appendix is surrounded by the omentum following perforation of acute appendicitis. The cases usually present with abdominal pain, nausea, vomiting, and abdominal mass. Chronic diarrhea is a rare finding. In this study, we explore a case of a 63-year-old man who had diarrhea and intermittent abdominal pain for 3 months and underwent a diagnosis of plastron appendicitis as a result of the investigations.

## Introduction

 Plastron appendicitis is an abscess formation that occurs when the appendix is surrounded by the omentum following perforation of acute appendicitis (1). The cases usually present with abdominal pain, nausea, vomiting, and abdominal mass. Chronic diarrhea due to plastron appendicitis is a very rare clinical condition. A limited number of cases were mentioned in the literature. In this case report, a case who applied with chronic diarrhea and intermittent abdominal pain and diagnosed as plastron appendicitis is discussed. 

## Case Report

A 63-year-old male patient was admitted to our clinic with diarrhea and intermittent abdominal pain for 3 months. The patient had mucoid-runny diarrhea 3-4 times a day. He had visceral pain at periumbilical region relieving with diarrhea. He had a history of gastrointestinal hemorrhage due to peptic ulcer 10 years ago. He did not have any history of drug use or operation. On physical examination; fever was 36 ° C, pulse was 82/min, arterial blood pressure was 130/80 mmHg. He had abdominal distention, increased bowel sounds and ileocecal tenderness with deep palpation. Other physical examination findings were normal. He did not have fever on the follow-up. Abnormal laboratory findings were white blood cell: 14,000/uL, C-reactive protein: 185 mg/L, erythrocyte sedimentation rate: 60 mm/h. Celiac markers were negative. Many leukocytes were seen in the stool microscopy. No parasitic organism was identified. In stool culture, no specific microorganism was detected. The patient underwent gastroscopy and colonoscopy. During the colonoscopy, purulent fluid was evacuated. Ulcerated and granular appearance was detected on caecum base, around the appendix orifice. Colitis was detected in the examination of the biopsy taken from this area. The patient's abdominal pain did not regress with conservative treatment. The patient's complaints could not be explained and abdominal imaging was planned. Inflammation of intestinal loops, omental inflammatory thickening and 7x5x4 cm fluid collection in pericaecal area were observed on abdominal ultrasonography (USG). Abdominal CT revealed a hyperdense area of 12x16 mm in heterogeneous structure within the anterior mesenteric fat tissue at the ileocecal valve level. It was found to be compatible with plastron appendicitis. 

The patient was hydrated and taking empiric cefixime 1x100 mg metronidazole 3x500 mg. His pain was decreased by drainage of purulent fluid, antibiotics and hydration. C-reactive protein was reduced to 19 mg/L. White blood cell count was 9,000/μL after treatment. The patient was discharged and called to the outpatient clinic 6 weeks later. Elective appendectomy operation was performed 6 weeks later. 

## Discussion

Diarrhea is an increase in the frequency of defecation and the rate of fluid in the feces as a result of impairment of intestinal absorption and secretion function (2). Chronic diarrhea is diarrhea that lasts for four weeks (3). Malabsorption syndromes especially celiac disease, inflammatory bowel diseases, irritable bowel disease, motility disorders, chronic parasitic infections, bacterial toxins, drugs, osmotic diarrhea and laxative use are considered as the causes of chronic diarrhea (4). Stool microscopy, parasite investigations, biopsy for inflammatory bowel disease with rectosigmoidoscopy and specific tests for malabsorption syndromes are performed in diarrhea that lasts more than four weeks (5). Plastron appendicitis that occurs due to appendix perforation is an acute condition which is ignorable while the investigations are performed for chronic diarrhea. A case of plastron appendicitis with a complaint of diarrhea for 3 months was discussed in this report. He was diagnosed with plastron appendicitis which can be ignored during investigating for the causes of chronic diarrhea.

As a result, plastron appendicitis is among the causes of chronic diarrhea. It should be kept in mind as a differential diagnosis during investigating the causes of chronic diarrhea. Abdominal imaging is needed to be performed for diagnosis in suspected patients. Antibiotic therapy and hydration should be provided and elective appendectomy should be planned.

## Conflict of interests

The authors declare that they have no conflict of interest.
